# Periplasmic methionine sulfoxide reductase (MsrP)—a secondary factor in stress survival and virulence of *Salmonella* Typhimurium

**DOI:** 10.1093/femsle/fnad063

**Published:** 2023-07-04

**Authors:** Hari Balaji Chandra, Arijit Shome, Raj Sahoo, S Apoorva, Sanjeev Kumar Bhure, Manish Mahawar

**Affiliations:** Division of Biochemistry, ICAR-Indian Veterinary Research Institute, Izatnagar, Bareilly, Uttar Pradesh 243122, India; Division of Biochemistry, ICAR-Indian Veterinary Research Institute, Izatnagar, Bareilly, Uttar Pradesh 243122, India; Division of Biochemistry, ICAR-Indian Veterinary Research Institute, Izatnagar, Bareilly, Uttar Pradesh 243122, India; Division of Biochemistry, ICAR-Indian Veterinary Research Institute, Izatnagar, Bareilly, Uttar Pradesh 243122, India; Division of Biochemistry, ICAR-Indian Veterinary Research Institute, Izatnagar, Bareilly, Uttar Pradesh 243122, India; Division of Biochemistry, ICAR-Indian Veterinary Research Institute, Izatnagar, Bareilly, Uttar Pradesh 243122, India

**Keywords:** Oxidants, Methionine oxidation, methionine sulfoxide reductase, periplasmic Msr, protein repair, *S*. Typhimurium

## Abstract

Among others, methionine residues are highly susceptible to host-generated oxidants. Repair of oxidized methionine (Met-SO) residues to methionine (Met) by methionine sulfoxide reductases (Msrs) play a chief role in stress survival of bacterial pathogens, including *Salmonella* Typhimurium. Periplasmic proteins, involved in many important cellular functions, are highly susceptible to host-generated oxidants. According to location in cell, two types of Msrs, cytoplasmic and periplasmic are present in *S*. Typhimurium. Owing to its localization, periplasmic Msr (MsrP) might play a crucial role in defending the host-generated oxidants. Here, we have assessed the role of MsrP in combating oxidative stress and colonization of *S*. Typhimurium. Δ*msrP* (mutant strain) grew normally in *in-vitro* media. In comparison to *S*. Typhimurium (wild type), mutant strain showed mild hypersensitivity to HOCl and chloramine-T (ChT). Following exposure to HOCl, mutant strain showed almost similar protein carbonyl levels (a marker of protein oxidation) as compared to *S*. Typhimurium strain. Additionally, Δ*msrP* strain showed higher susceptibility to neutrophils than the parent strain. Further, the mutant strain showed very mild defects in survival in mice spleen and liver as compared to wild-type strain. In a nutshell, our results indicate that MsrP plays only a secondary role in combating oxidative stress and colonization of *S*. Typhimurium.

## Teaser

Periplasmic Msr mutant (Δ*msrP*) of *Salmonella* Typhimurium strain does not show growth defect in various *in vitro* media like LB and M9 broth media as compared to the wild-type strain (Msr–Methionine sulfoxide reductase).

## Introduction

Typhoidal and non-typhoidal *Salmonella* (NTS) are the major etiological agents of gastro-intestinal diseases in humans. *Salmonella* is associated with more than 100 million annual cases of human infections worldwide (Sears et al. 2021). Typhoidal *Salmonella* is generally restricted to human and include serovars like *S. enterica* Typhi and *S. enterica* Paratyphi, which cause enteric fever (Bula-Rudas et al. 2015). NTS includes *S. enterica* serovar Typhimurium (*S*. Typhimurium) and *S. enterica* serovar Enteritidis (*S*. Enteritidis) (Gal-Mor et al. 2014). NTS-associated infection is common in old and immunocompromised individuals (Gordon 2008) and is estimated to cause over 90 million cases and 100 000 annual deaths worldwide (Majowicz et al. 2010). Interestingly, *S*. Typhimurium accounts for about 21.9% of cases of poultry salmonellosis in India (Kumar et al. 2019). Most of the infected poultry birds serve as asymptomatic reservoirs of NTS. However, poultry products are one of the major sources of NTS infection in humans (Ferrari et al. 2019).

Inside the host, *S*. Typhimurium experiences several stresses, including extreme pH of gastric and intestinal secretions, antimicrobial peptides, and various reactive oxygen, chlorine, and nitrogen species (ROS, RCS, and RNS). *Salmonella*, being an intracellular pathogen, can survive and replicate inside phagocytic cells. Phagocytes generate various ROS, RCS, and RNS which can damage bacterial biomolecules, including DNA, proteins, and lipids (Mastroeni et al. 2000). RCS molecules like HOCl are generated during respiratory burst and are highly reactive with methionine residues. To survive in such adverse environment of phagocytic cells, *Salmonella* has evolved a wide range of strategies. SPI-2 encodes an effector protein SpiC, which prevents the assembly of phagosomal oxidase and improves the survival of *S*. Typhimurium in *Salmonella*-containing vacuoles (SCV) (Vazquez-Torres and Fang 2001). Primary antioxidant enzymes like catalases, superoxide dismutases, and alkyl hydroperoxides catalytically act on the host-generated **oxidants like** H_2_O_2_, O_2_^• −^, ONOO^•^, respectively (Hébrard et al. 2009, Kröger et al. 2013).

During respiratory burst, the oxidants generated by immune cells exceed the scavenging capacities of primary anti-oxidant enzymes, which results in oxidative modifications of different biomolecules. Possessing electron rich sulphur, cysteine, and methionine residues are highly susceptible to oxidation (Bin et al. 2017). After oxidation, methionine (Met) residues convert into methionine sulfoxide (Met-SO) (either *R*- or *S*-form) (Stadtman et al. 2005). Oxidation of Met residues can alter protein structure, function, and consequently affect cellular viability. However, methionine sulfoxide reductases (Msrs), with the help of thioredoxin and thioredoxin reductase can reduce Met-SO to Met and thus improve the bacterial survival under oxidative stress conditions (Zhang and Weissbach 2008).

Based on location, stereospecificity, and specificity to peptide bound or free Met-SO, four cytoplasmic Msrs have been identified in *S*. Typhimurium, namely MsrA, MsrB, MsrC, and BisC. MsrA is specific for *S*-form of Met-SO and can act on both free or peptide-bound Met-SO residues (Denkel et al. 2011). MsrB is highly specific for *R*-form of Met-SO and is active only on peptide-bound Met-SO residues. MsrC and BisC act on free *R*- and *S*-forms of Met-SO, respectively (Denkel et al. 2011). Cytoplasmic Msrs have been shown to alleviate oxidative stress and enhance the survival of *S*. Typhimurium under oxidative stress. Δ*msrA*, Δ*msrC*, and Δ*bisC* mutants of *S*. Typhimurium were shown to be hypersusceptible to oxidants and phagocytic cells. Further, these strains showed defective colonization in mice and poultry (Denkel et al. 2011, Denkel et al. 2013, Trivedi et al. 2015, Sarkhel et al. 2017, Nair et al. 2021). However, deletion of *msrB* gene does not affect the viability of *S*. Typhimurium against oxidants and colonization in mice (Denkel et al. 2011).

Owing to its location, periplasmic compartment first comes into contact with host-generated ROS/RNS and thus Met residues in periplasmic proteins are highly vulnerable to oxidation. Therefore, Msrs are required in extracytoplasmic space to repair Met-SO residues in oxidized proteins. Indeed, extracytoplasmic Msrs have been identified in different bacterial species, which highlights the importance of the repair of oxidized proteins in this compartment. Most of these extracytoplasmic Msrs are fusion proteins, containing MsrA and MsrB domains. PilB, a fimbrial protein in *Neisseria gonorrhoeae*, was the first extracytoplasmic protein identified to have Msr activity (Olry et al. 2002). Extracytoplasmic Msrs (MsrAB) in *S. pneumoniae* and *H*. influenzae contribute to survival of these bacterial pathogens against oxidative stress (Saleh et al. 2013, Nasreen et al. 2020).

In *Escherichia coli*, unlike the above extracytoplasmic Msrs, a novel periplasmic Msr (MsrP) has been identified. MsrP, along with periplasmic membrane redox protein (MsrQ) forms MsrPQ system (Gennaris et al. 2015). MsrP can reduce both *R-* as well as *S*-forms of l-Met-SO and have catalytic activity on both free and peptide-bound l-Met-SO (Gennaris et al. 2015, Tarrago et al. 2018). MsrP system has been shown to be required for survival against HOCl stress in *Escherichia coli* (Gennaris et al. 2015) and nitrosative stress in *Campylobacter jejuni* (Hitchcock et al. 2010). A recent study has identified MsrP system in *S*. Typhimurium which can reduce both free and peptide bound, as well as, *R*- and *S*-forms of Met-SO (Andrieu et al. 2020). MsrP might play a very important role in oxidative stress survival of *S*. Typhimurium. However, the contribution of MsrP in the survival of *S*. Typhimurium under oxidative stress and in virulence is not known.

In this study, we have assessed the contribution of MsrP under oxidative stress and colonization of *S*. Typhimurium.

## Materials and methods

All animal experiments were approved by the Institutional Animal Ethics Committee (IAEC), Indian Council of Agricultural Research-Indian Veterinary Research Institute (ICAR-IVRI), Izatnagar, India. All animal experimentations were performed in accordance with the ARRIVE guidelines.

### Bacterial strains and plasmids

*Salmonella enterica* subspecies *enterica* serovar Typhimurium strain E-5591 (*S*. Typhimurium) was procured from the National *Salmonella* Center, ICAR-IVRI, Izatnagar, India. The NEB-5α strain of *E*. coli was purchased from New England BioLabs. The plasmids pKD3, pKD46, and pCP20 plasmids were generously provided by Prof. Robert J. Maier, UGA, Athens, GA, USA.

*Salmonella* was cultured in Hektoen enteric (HE) agar or Luria Bertani (LB) broth. Growth media were supplemented either with chloramphenicol (Cm) (20 µg/ml), kanamycin (Kan) (50 µg/ml), or ampicillin (Amp) (100 µg/ml) for selection purposes as and when required.

### Generation of Δ*msrP* mutant strain in *S*. Typhimurium

**Figure 1.**
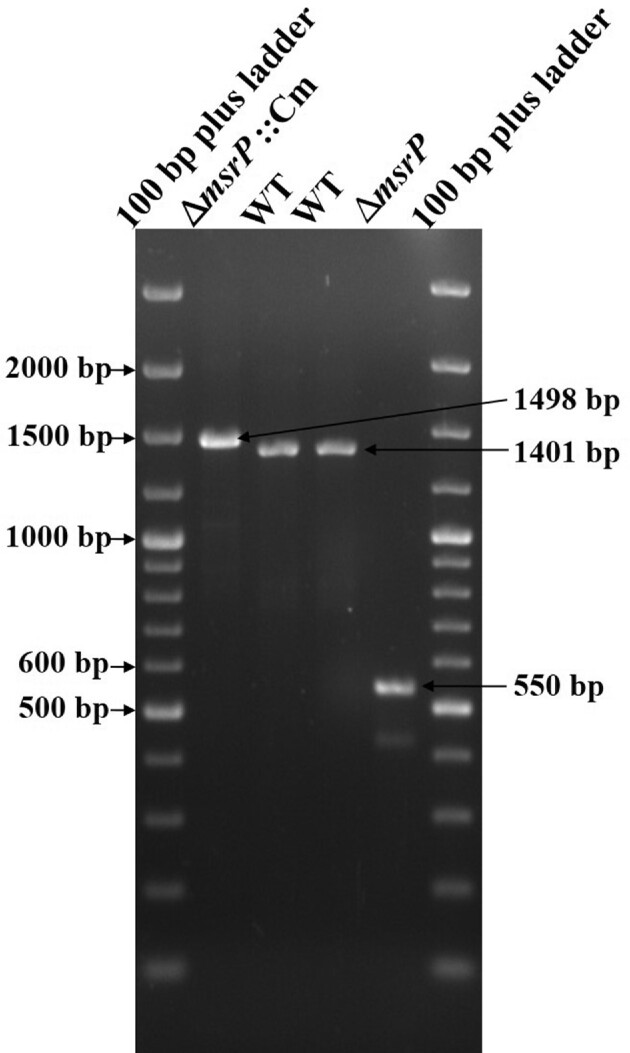
Construction of *msrP* gene deletion (∆*msrP*) strains in *S*. Typhimurium. Agarose gel analysis of different steps for *msrP* mutant construction. The *msrP* gene was deleted as described in materials and methods and confirmed by PCR. Various templates used for PCR reactions are labeled on the top of various lanes.

The *msrP* gene deletion strain (Δ*msrP* mutant strain) was constructed using lambda red recombinase-mediated gene inactivation protocol (Datsenko and Wanner 2000). Briefly, FRT flanked Cm cassette was amplified from pKD3 plasmid by using MsrP_New del For and MsrP_New del Rev primers (Table 1). The PCR conditions are detailed in Table 1. The purified Cm cassette was transformed into lambda red recombinase expressing *S*. Typhimurium. Cm-supplemented agar media was used to select recombinants and confirmed by PCR (Fig. 1). The Cm cassette was then removed by flip recombinase (Datsenko and Wanner 2000).

**Table 1.** Primers used in the study and their PCR conditions.

Sl No	Primer Name	Sequence	PCR conditions	Product size	Purpose	Reference
1	MsrP_ New del For.	5′GACCGGGAGTCTGTGA TGAAAAAGATACGTCCA TTAACAGAAGCCGTGTA GGCTGGAGCTGCTTC 3′	\begin{eqnarray*} \begin{array}{@{}l@{}}{\rm 95 ^\circ C\ \ 5\, min}\\\begin{array}{@{}l@{}} {\rm 95 ^\circ C\ \ 30\, s}\\ {\rm 62 ^\circ C\ 40\, s}\\{\rm 72 ^\circ C\ 100\, s} \end{array}\Big \} \times 35\\{\rm 72 ^\circ C\ \ 10\, min} \end{array} \end{eqnarray*}	1092 bp	To amplify FRT flanked Chloramphenicol cassette	Current study
2	MsrP_New del Rev.	5′TGCTGTCAGACGCACT TAAAAATTCTCCCGCAA ATTGAGACCGCGCATAT GAATATCCTCCTTAG 3′				
3	MsrP ST test For.	5′AGGGCCGTACGCTGGT GAAGAT 3′	\begin{eqnarray*} \begin{array}{@{}l@{}} {\rm 95 ^\circ C\ \ 5\, min}\\ \begin{array}{@{}l@{}} {\rm 95 ^\circ C\ \ 30\, s}\\ {\rm 62 ^\circ C\ 40\, s}\\{\rm 72 ^\circ C\ 100\, s} \end{array}\Big \} \times 35\\ {\rm 72 ^\circ C\ \ 10\, min} \end{array} \end{eqnarray*}	550 bp in Δ*msrP* strain and 1401 bp in *S*. Typhimurium	Confirmation of *msrP* deletion	Current study
4	MsrP ST test Rev.	5′GAAACACCATAATCCT AACAGGCG 3′				

### *In vitro* growth evaluation

The mutation might affect *in vitro* proliferation of bacteria. To determine the effect of *msr* gene deletions on *in vitro* growth of *S*. Typhimurium, the growth of various strains were assessed in LB broth or M9 media. In brief, isolated colonies of different strains were grown in LB broth or M9 media overnight at 37°C. On the following day, overnight grown cultures were diluted in 50 ml fresh media @ 1:100 ratio and incubated in a shaker incubator at 37°C. Aliquots of 1 ml were taken at hourly intervals and the absorbance was recorded at 600 nm (Nair et al. 2021).

### HOCl and chloramine-T susceptibility assays

The susceptibility of wild-type and mutant *S*. Typhimurium strains to HOCl (NaOCl, Sigma) and chloramine-T (ChT) (N-Chloro-*p-*toluenesulfonamide trihydrate sodium salt, Sigma) was assessed as described earlier (Nair et al. 2021). In brief, the vigorously growing cultures of wild-type and mutant strains was pelleted, washed and suspended in PBS at an OD_600nm_ of 1.0. The suspensions were then treated with various concentrations of HOCl (50 and 100 µM) or ChT (100, 200, 300, 400, and 500 µM) for 30 min. After 30 min of exposure, the mix was supplemented with 10 mM (final) l-methionine and incubated for 15 min. The suspensions were then serially diluted and plated on HE agar plates. The plates were incubated at 37°C and colonies were counted (Trivedi et al. 2015, Nair et al. 2021).

### Quantification of total protein carbonyls

Total protein carbonyls of whole cellular proteome were determined using DNPH assays as described earlier (Apoorva et al. 2020). Shortly, 0 and 3 mM HOCl-exposed cultures of wild-type and mutant strains were pelleted. The pellets were suspended in BugBuster^TM^ Protein Extraction Reagent and incubated for 30 min at room temperature, with intermittent shaking at every 5 min. After incubation, the cell debris was removed by centrifugation at 15 000 × *g* for 30 min at 4ºC. The supernatants were collected and incubated with 600 µl of 10 mM DNPH (in 2.5 M HCl) for an hour in dark, with intermittent vortexing at every 15 min. The proteins were precipitated by addition of 10% TCA (final) and collected by centrifugation at 15 000 × *g* for 20 min at 4ºC. Pellets were then washed twice with 10% TCA and once with ethanol and ethylacetate (1:1). The final precipitate was dissolved in 6 M guanidine hydrochloride and incubated for 30 min at 37ºC, with intermittent vortexing. The total protein carbonyls were determined by recording the absorbance at 355 nm using 6 M guanidine HCl as blank. Carbonyl levels were calculated using the formula: A_355_/(εl), where ε = 22 000 M^−1^ cm^−1^ (molar absorption coefficient of hydrazone is 22 000 M^−1^ cm^−1^), c = concentration of carbonyls in moles/litre (M), and A = absorbance at 355 nm (Reznick and Packer 1994). The total protein carbonyls were expressed as nmol/mg of proteins.

### Neutrophil sensitivity assays

The sensitivity of different strains of *S*. Typhimurium to neutrophils was assessed as described elsewhere (Okamura and Spitznagel 1982, Oh et al. 2008) with minor modifications. Briefly, goat blood (collected in EDTA) was diluted with an equal quantity of PBS. The diluted blood was then layered over a mixture of equal volumes of Histopaque 1077 and 1119 and centrifuged at 750 × *g* at room temperature for 45 min. Neutrophils present at the interface point between the Histopaque layers were pipetted out and washed twice with PBS by centrifuging at 250 × *g* for 10 min. A final wash was done with HBSS without Ca^2+^ and Mg^2+^ and the neutrophils were suspended in the same medium. The vigorously growing cultures of various *Salmonella* strains were pelleted, washed, and suspended in HBSS with Ca^2+^ and Mg.^2+^ Bacteria and neutrophils were mixed in a ratio of 10:1 and incubated in a CO_2_ incubator at 37°C, for 60 min. Post incubation, the mix was centrifuged at 13 000 rpm for 3 min. The supernatant was discarded, the pellet was treated with 0.1% Triton X-100 (final concentration) and finally, the lysates were diluted and plated on HE agar. Plates were incubated at 37°C and colonies were enumerated.

### Competitive infection assays in mice

The effect of deletion of the *msrP* gene in the virulence of *S*. Typhimurium was assessed in Swiss Albino mice.

In brief, the overnight grown cultures of *S*. Typhimurium strain and Δ*msrP*::Cm strains were diluted in 50 ml LB broth and grown upto an OD_600nm_ of 0.80. The cultures were then pelleted and suspended in PBS to obtain CFUs of 4 × 10^4^/50 µl. The suspensions of two strains were mixed at 1:1 ratio and a total of 100 µl containing 8 × 10^4^ CFUs were inoculated in each mouse by intra-peritoneal route. Actual bacterial numbers were determined by retrospective plating. The mice were sacrificed following 3- and 5-days post-infection and the spleen and liver were homogenized in PBS. The homogenates were diluted and plated on plain as well as antibiotic-containing agar media (HE agar for *S*. Typhimurium and HE agar supplemented with Cm for Δ*msrP* strain).

Competitive index (CI) was calculated as described elsewhere (Denkel et al. 2011, Kumawat et al. 2016). CI is the ratio of mutant to wild-type strain recovered divided by the ratio of mutant to wild-type strain inoculated.

## Results

### MsrP is not essential for *in vitro* growth of *S*. Typhimurium

**Figure 2.**
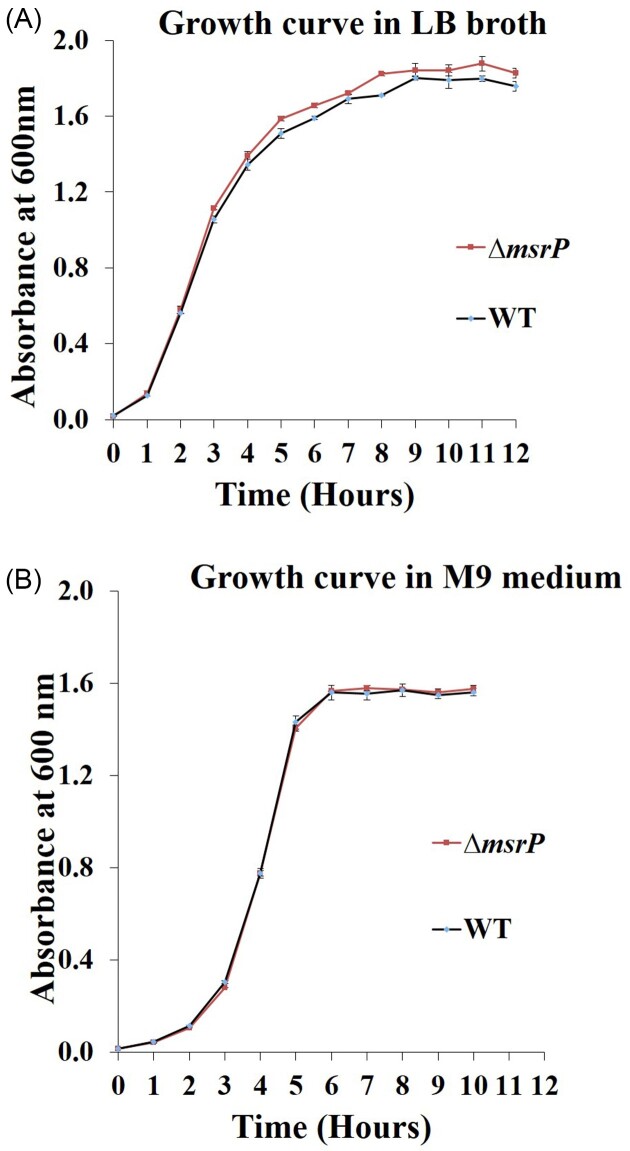
**∆** *msrP* strain does not show growth defect in various *in vitro* media. The WT and ∆*msrP* strains of *S*. Typhimurium were cultured in LB **(2A)** or M9 minimal medium **(2B)**. Aliquots were withdrawn at various times post inoculation and absorbance were recorded at 600 nm. Data are presented as mean ± S. D. (*n* = 3).

The growth of wild-type and mutant strains was assessed for duration of 12 hours in LB broth and M9 media. In both the media, sigmoidal-shaped growth curves were observed in wild-type and mutant strains. The results indicate that the *in vitro* growth of mutant strain was almost similar to the wild-type counterpart (Fig. 2).

### Δ*msrP* strain shows very mild hypersensitivity to oxidants (HOCl and ChT)

**Figure 3.**
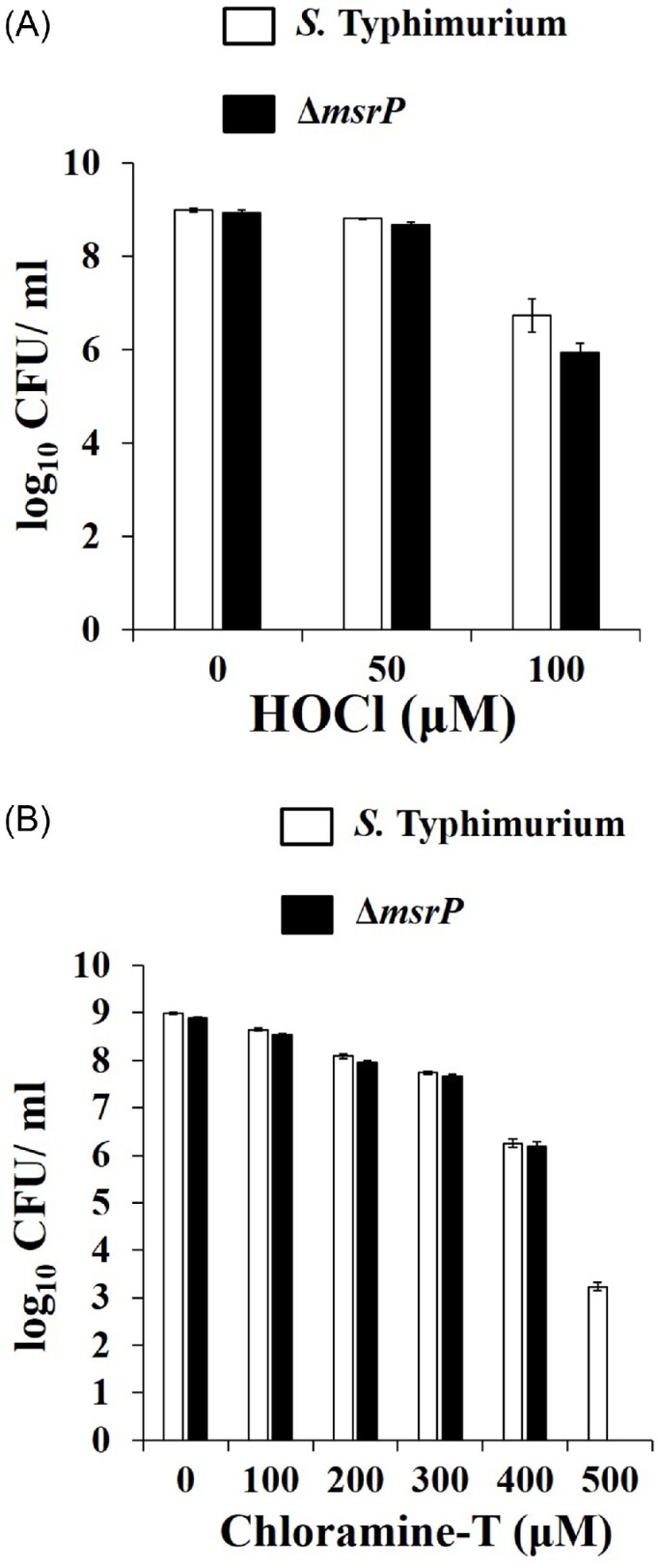
Oxidant susceptibility assays. *S*. Typhimurium and ∆*msrP* strains were grown in LB broth, pelleted, suspended in PBS, and exposed to indicated concentrations of HOCl (3A) or ChT (3B) for 30 min. Excess oxidants were then quenched by addition of l-methionine. The cultures were then serially diluted and plated on HE agar plates. Following overnight incubation, the colonies were counted and expressed as log_10_ CFU per ml. Data are presented as mean ± SE (*n* = 4) for HOCl; (*n* = 5) for ChT.

Next, we determined the effect of deletion of the *msrP gene* on survival of *S*. Typhimurium against HOCl and ChT. In comparison to the wild-type, the mutant strain was slightly (7.7 folds) (*P* = 0.0612), but not significantly sensitive to 100 µM HOCl (Fig. 3A). Mutant strain showed almost similar sensitivity to 100–400 µM ChT as shown by wild type (Fig. 3B). Following incubation with 500 µM concentration of ChT, we recovered few colonies in *S*. Typhimurium only, indicating significant sensitivity of the mutant strain (*P* < 0.0001) only at this concentration.

### Δ*msrP* strain shows analogous levels of protein carbonylation under oxidative stress

**Figure 4.**
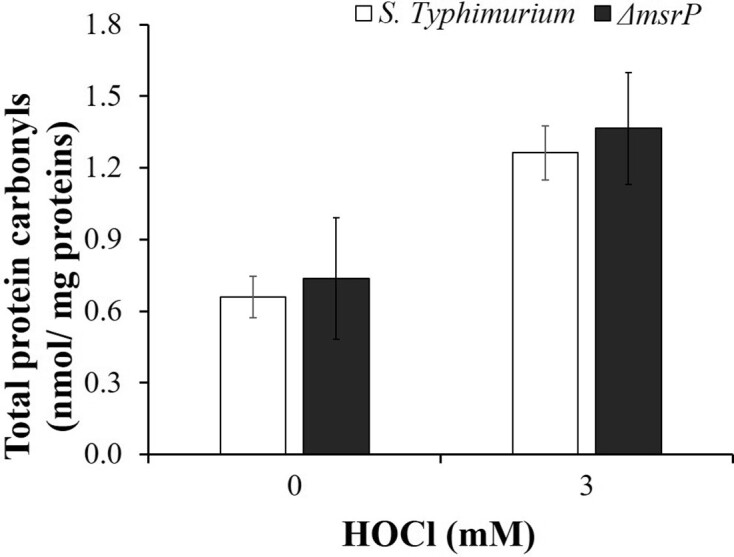
Estimation of total protein carbonyl contents of whole proteome in various strains of *S*. Typhimurium. Cell free lysates of 0 and 3 mM HOCl-exposed cultures were derivatized with 10 mM 2, 4-DNPH. Protein carbonyl levels were determined as described in the section ‘Materials and Methods’. The data are presented as mean ± SE (*n* = 3).

Carbonyls are considered as stable markers of protein oxidation (Dalle-Donne et al. 2003) and can be exploited to assess the levels of protein oxidation as well as relative cellular susceptibility to oxidants. We quantified the total protein carbonyl levels of the whole proteome following exposure of *S*. Typhimurium and Δ*msrP* strains to HOCl. Following HOCl exposure, Δ*msrP* strain showed almost similar levels (1.08-folds) of protein carbonyls as that of *S*. Typhimurium (Fig. 4).

### Δ*msrP* strain exhibited hypersensitivity to neutrophils

**Figure 5.**
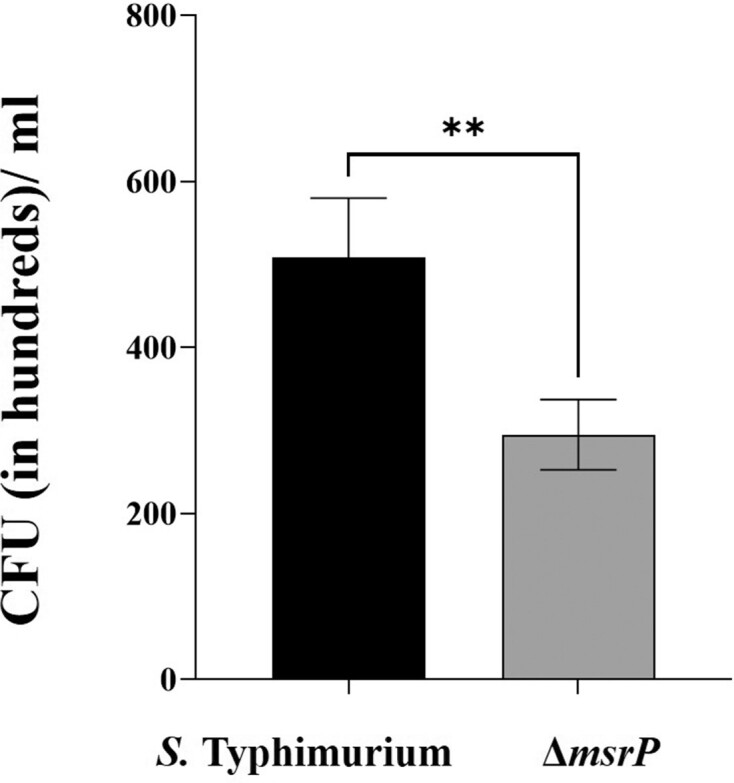
Neutrophil sensitivity assays. Different strains of *S*. Typhimurium were treated with neutrophils at a ratio of 10:1 (bacteria: cells) for 60 min. Following incubation, the mix was centrifuged and the pellet was treated with 0.1% Triton-X 100. The lysates were then serially diluted and plated on HE agar. CFUs were counted following overnight incubation of the plates. The data are presented as mean ± SD (*n* = 6) and the experiment was repeated twice. The data were analysed by paired *t*-test. **P ≤ 0.001.

The recovered numbers of wild-type and mutant strains following incubation with neutrophils were (CFUs/ml in hundreds, mean ± SD) 508.33 ± 71.39 and 295 ± 42.30, respectively. As compared to the wild-type, mutant strain was 1.72-folds (*P* < 0.001) more susceptible to neutrophils (Fig. 5).

### The fitness of Δ*msrP* strain is not compromised in mice spleen and liver

A separate study demonstrated that the fitness of Δ*msrA*, but not of Δ*msrB* strain of *S*. Typhimurium is compromised in mice (Denkel et al. 2011). However, the contribution of *msrP* gene on colonization in mice is not known.

We evaluated the fitness of Δ*msrP* strain in comparison with *S*. Typhimurium, by inoculating a mixture of both *S*. Typhimurium and Δ*msrP* strains in the mice. The CI values are depicted in Table 2. Following 3- and 5-days post-infection, we recovered slightly higher numbers of wild type than that of mutant strain from spleen and liver.

**Table 2.** CI of Δ*msrP* strain vs *S*. Typhimurium in mice.

Days Post Infection	CI (mean ± SE)	
	Spleen	Liver
3	0.50 ± 0.12 (*n* = 5)	0.47 ± 0.13 (*n* = 5)
5	0.96 ± 0.26 (*n* = 5)	0.46 ± 0.14 (*n* = 5)

## Discussion

Intracellular pathogens like *S*. Typhimurium constantly encounter oxidants during colonization in the host (Noster et al. 2019). Met residues are easy targets of oxidation, affecting the protein function. The protease-mediated degradation followed by ribosomal synthesis is the usual way to restore protein function in the cell. However, the repair of oxidized proteins by Msrs is an energy-efficient approach to maintain the functional protein pool in the cell. Thus, Msrs play a necessary role in the bacterial survival during the infection process, where they are exposed to copious amounts of oxidants.

Periplasm is a narrow space located between the outer and inner cell membrane it constitutes about 10%–20% of cell volume and is the home for about 367 proteins in *E. coli* (Weiner and Li 2008). On the other hand, periplasmic proteins, due to their localization, are the prime targets of host-generated oxidants. The oxidizing environment of periplasm further augments the oxidative damage to these proteins. Therefore, bacterial pathogens have evolved mechanism(s), which can protect periplasmic proteins from oxidative damage. Enzymes like DsbA catalyze the formation of disulfide bridges in periplasmic proteins and promote their stability (Depuydt et al. 2011).

Very recently a unique periplasmic Msr (MsrP) system has been identified (Gennaris et al. 2015). However, the contribution of MsrP in protection of the bacterial pathogens against oxidative stress and virulence has not been evaluated. MsrP along with MsrQ can reduce both free and protein-bound as well as *R-* and *S-* stereoisomers of Met-SO (Gennaris et al. 2015, Andrieu et al. 2020) in *E. coli* as well as in *S*. Typhimurium. *Salmonella* Met auxotroph mutant lacking all Msrs (Δ*metA* Δ*4msr^cyto^* Δ*msrP)* failed to grow on a media containing Met-SO as the sole source of methionine, suggesting that *S*. Typhimurium encodes only 5 Msrs (Andrieu et al. 2020). These findings lead us to hypothesize that Δ*msrP* strain might be highly susceptible to oxidative stress.

First, we have generated *msrP* gene deletion strain in *S*. Typhimurium. Subsequently, we have assessed the contribution of *msrP* in resisting oxidative stress *in vitro* and survival of *S*. Typhimurium *in vivo*. Whenever there are nutritional or environmental changes, bacteria adapt itself to new microenvironment and also upregulate the expression of genes required for growth during the lag phase (Vermeersch et al. 2019). Adapted cells then enter into the exponential phase, where cells divide at a constant rate (Navarro Llorens et al. 2010). However, steady growth and exhaustion of nutrients lead to a stationary phase where bacteria encounter ROS generated by aerobic respiration (Dukan and Nyström 1998). Indeed, *msrA* and *msrB* genes were found to be upregulated during stationary phase of growth in *S*. Typhimurium (Rolfe et al. 2012). First, we analysed the effect of deletion of *msrP* on the *in vitro* growth of *S*. Typhimurium. We observed that the growth of Δ*msrP* strain was almost similar to that of *S*. Typhimurium (Fig. 2). This indicated that deletion of *msrP* doesn't have an effect on *in vitro* growth of *S*. Typhimurium.

Neutrophils and, to a lesser extent, macrophages contain myeloperoxidase (MPO), the enzyme that catalyzes formation of hypohalous acids from H_2_O_2_ and halides (Winterbourn and Kettle 2013). Due to the abundance of Cl^−^ ions, the major product of the above reaction is HOCl (Ulfig and Leichert 2021). HOCl is a key powerful oxidant generated by host immune response (Klebanoff et al. 2013). Although H_2_O_2_ is more stable than HOCl, it is less reactive and can diffuse out of phagosomes faster (3 × 10^−3^ cm s^−1^), leading to collateral tissue damage. Due to its confined action inside phagosome, shorter half-life, and lesser diffusion rate, the production of HOCl is advantageous for host (Schürmann et al. 2017). Among the amino acids, cysteine and methionine are highly susceptible to oxidation by HOCl produced via the respiratory burst. Met residues were observed to have the highest reactivity to HOCl (Hawkins et al. 2003).

Mutants in cytoplasmic *msr*s of several bacterial pathogens like, *M. tuberculosis, E. coli*, and *P. aeruginosa* showed hypersensitivity to bleach stress (Lee et al. 2009, Rosen et al. 2009, Romsang et al. 2013). Interestingly, *S*. Typhimurium mutants lacking *msrA* or *msrAmsrC* (both genes together) genes were found to be hypersusceptible to HOCl (Trivedi et al. 2015, Nair et al. 2021).

In non-typeable *H. influenzae* (NTHi), MsrAB is localized in periplasm and is required for the survival of this bacterium against bleach stress (Nasreen et al. 2020). Similarly, a mutant strain lacking *msrP* gene in *E. coli* was found to be moderately susceptible to HOCl (Gennaris et al. 2015). On the other hand, the expression of *msrP* in *E. coli* and *msrAB* in NTHi was induced following HOCl exposure (Gennaris et al. 2015, Nasreen et al. 2020). A new study has found that MsrP does not have much impact on the survival of *S*. Typhimurium on exposure to HOCl in growing conditions (Andrieu et al. 2023).

In the current study, we also observed that Δ*msrP* strain showed only mild sensitivity to HOCl as compared to that of wild-type strain of *S*. Typhimurium in non-growing conditions (Fig. 3A).

*N*-chlorotaurine is a long-acting endogenous oxidizing agent produced mainly by activated granulocytes and monocytes (Eitzinger et al. 2012). During an oxidative burst, HOCl reacts with different amine groups and forms *N*-chloramines (Nagl et al. 2000). Within neutrophil phagosome, about 10%–50% of the HOCl reacts with free amines and results in generation of chloramines (Nagl et al. 2000). Since taurine accounts to more than half of the total amino acid pool in the neutrophils, *N*-chlorotaurine is the main product formed and also has more stability compared to other chloramines formed from α-amino acids (Gottardi and Nagl 2010). Although *N*-chlorotaurine is a poor oxidizing agent as compared to other N-Cl compounds, it acts as a vehicle for the strong oxidizing agent like HOCl during the oxidation burst, thus prolonging the oxidizing effect on pathogens (Anich et al. 2021). In a recent study, it has been shown that Δ*msrP* strain of *S*. Typhimurium shows poor survival on exposure to *N*-chlorotaurine in growing conditions. The same study established that *N*-chlorotaurine induces expression of MsrP in *S*. Typhimurium, elucidating the necessity of this periplasmic reductase during envelope stress (Andrieu et al. 2023). Similarly, we assessed the effects of deletion of *msrP gene* on survival of *S*. Typhimurium against ChT under non-growing condition. ChT is a structural analogue of *N*-chlorotaurine and preferentially oxidizes Met residues (Mahawar et al. 2011). Following incubation with ChT, mutant strain showed mild susceptibility as compared to that of wild-type strain of *S*. Typhimurium (Fig. 3B).

As discussed earlier, since proteins are the major target of oxidative damage and likewise many amino acid residues also undergo oxidative modifications (Hawkins and Davies 2019). One such modification is carbonylation. Carbonylation is the formation of aldehyde, ketone, or lactam in amino acid residues (Fedorova et al. 2014). Since carbonyl groups are introduced very early during protein oxidation and are relatively stable as compared to other modifications, they can be exploited to estimate extent of oxidative damage in proteins (Dalle-Donne et al. 2003). Mice lacking *msr* genes showed high levels of protein carbonyls in various tissues following oxidative stress (Moskovitz et al. 2001, Moskovitz and Stadtman 2003). Similarly, *msr* inactivated or deletion mutants of pathogens like *H. pylori* and *S*. Typhimurium showed increased protein carbonylations (Alamuri and Maier 2004, Denkel et al. 2011). Next, we have assessed the total protein carbonyl levels in different strains of *S*. Typhimurium. Following exposure to HOCl, almost identical levels of protein carbonyls were observed in mutant strain when compared to that of the wild-type strain (Fig. 4).

Neutrophils play a very important role in host defense against *Salmonella* infection (Brinkmann et al. 2004) and are the key cells involved in spread of *S*. Typhimurium. Neutrophils use various mechanisms to kill invading bacteria (Geddes et al. 2007). These mechanisms include, formation of neutrophil extracellular traps (NETs), secreting cytokines, releasing proteases, and generation of ROS (Liew and Kubes 2019). Due to the presence of Nox system (source of superoxide ion) and MPO system (source of HOCl), neutrophils produce copious amounts of ROS and RCS (Segal 2005, Davies and Hawkins 2020). Indeed, MPO-deficient mice were shown to be hypersusceptible to *Salmonella* infection (Burton et al. 2014). Depletion of neutrophils using anti-RB6–8C5 antibodies resulted in enhanced *Salmonella* infection in mice (Vassiloyanakopoulos et al. 1998). *Salmonella* deploys various strategies to survive inside the neutrophils. One of them is a repair of its oxidized proteins by Msrs (Cheminay et al. 2004, Westerman et al. 2021). Indeed, *S*. Typhimurium mutant lacking *msrA* was shown to have poor survival inside neutrophils (Trivedi et al. 2015).

To assess the degree of importance of periplasmic Msr in the survival of *S*. Typhimurium inside neutrophils, we performed the neutrophil sensitivity assays. Curiously, Δ*msrP* mutant strain (*P* < 0.001) was found to be highly sensitive to neutrophils in comparison to *S*. Typhimurium (Fig. 5).

Few studies used *msr* gene deletion strains of *S*. Typhimurium to elaborate on the importance of Msrs in *in vivo* colonization in mice (Denkel et al. 2011, Denkel et al. 2013). The fitness of Δ*msrA* and Δ*msrC* strains but not of Δ*msrB* strain has been shown to be compromised in mice (Denkel et al. 2011). Studies pertaining to importance of periplasmic msr in *in vivo* survival are scarce. A mutant lacking *cj0379c* gene (periplasmic msr) in *C. jejuni* was shown to have defective colonization in poultry (Hitchcock et al. 2010). Interestingly, our results suggest that the *msrP* plays only a minor role in mice colonization (Table 2).

In *E. coli*, the enhanced expression of *msrP* was observed under bleach stress. Further, MsrP was found to protect the activity of periplasmic chaperones like SurA (Gennaris et al. 2015). However, our results suggest that the role of MsrP might be secondary to cytoplasmic Msrs in maintaining the cell viability under stress conditions. Few studies suggested the possibility of relatively high resistance of periplasmic proteins to oxidative damage and aggregation following oxidative and chemical stress. A study reported that when periplasmic proteins of *E. coli* were subjected to various stresses (heat stress, 60% ethanol, 0.5 M HCl, or 5 mM CuSO_4_), they tend to stay soluble and aggregate less in comparison to that of non-periplasmic proteins (Liu et al. 2004). Another study observed that exposure of *E. coli* to HOCl resulted in the oxidation of Met residues in both periplasmic and cytoplasmic proteins. However, the study showed that the bacterial death is linearly proportional to Met oxidation in periplasmic proteins, only after 30%–40% of Met in periplasmic proteins were oxidized. In contrast to that, amount of Met oxidation in cytoplasmic and inner membrane proteins is always linearly proportional to bacterial killing, with complete bacterial death even at 10%–35% met oxidation in cytoplasmic proteins (Rosen et al. 2009).

Many Gram-negative bacteria have evolved to restrict the entry of harmful substances by altering their membrane permeability (Martinez et al. 2001). Indeed, it was reported that in *S*. Typhimurium, permeability to H_2_O_2_ is highly modulated by two outer membrane proteins (OMPs)—OmpA and OmpC. Interestingly, OmpA was reported to have a periplasmic domain with two specific cysteine residues whose oxidation status determines the opening and closing of this porin (van der Heijden et al. 2016). However, similar mechanisms to alter membrane permeability to HOCl and n-chlorotaurine in Gram-negative bacteria as a possible way to deter oxidation of membrane proteins need to be assessed.

In summary, our study suggests that periplasmic Msr plays a relatively insignificant role in combating oxidative stress and virulence of *S*. Typhimurium. Our findings indicate that MsrP could act as a secondary line of defence against bleach stress by indirectly quenching the incoming HOCl and chloramines from oxidizing the more susceptible cytoplasmic proteins.

## Data Availability

All data generated or analysed during this study are included in this published article.
